# Psychological distress in French college students: demographic, economic and social stressors. Results from the 2010 *National Health Barometer*

**DOI:** 10.1186/1471-2458-14-256

**Published:** 2014-03-17

**Authors:** Thomas Saïas, Enguerrand du Roscoät, Laurentine Véron, Romain Guignard, Jean-Baptiste Richard, Stéphane Legleye, Fanny Sauvade, Viviane Kovess, François Beck

**Affiliations:** 1 Institut National de Prévention et d’Education pour la Santé, 42 Bd de la Libération, Saint-Denis, Cedex 93203, France; 2 Département de Psychologie, Université du Québec à Montréal, Montréal, Québec, Canada; 3 EA 4386 - Laboratoire Parisien de Psychologie Sociale (LAPPS), Université Paris Ouest Nanterre-La Défense, Nanterre, France; 4 Aspytude, 20 cours Vitton, Lyon 69006, France; 5 INED, 133 boulevard Davout, Cedex 20 75980, Paris; 6 Département Epidemiologie et Biostatistiques EHESP, EA4069 Université Paris Descartes SPC, Avenue du Professeur Léon-Bernard CS 74312, Rennes 35043, Paris; 7 Cermes3 - Equipe Cesames (Centre de recherche Médecine, Sciences, Santé, Santé mentale, Société, Université Paris Descartes, Sorbonne Paris Cité/CNRS UMR 8211/Inserm U988/EHESS), Paris, France

**Keywords:** Psychological distress, Students, Risk factors, Social isolation, Prevention

## Abstract

**Background:**

Psychological distress (PD) in students is under-investigated, since its prevalence can be high in certain subgroups of students and it has been seen to be associated with other mental health issues and academic achievement. In a sample of French college students, this study investigated factors associated with PD, and looked more closely at the impact of social and interpersonal variables.

**Methods:**

Data were extracted from the 2010 French “National Health Barometer”. 946 students were interviewed. Mental health was assessed using the MH-5 five-item scale.

**Results:**

The PD rate in this sample was 13.8% (7.2% in males, 19.5% in females). Low income, nonsexual assault in the last 12 months, studying law and low social participation were associated with PD in multivariate analyses.

**Conclusions:**

French students show specific characteristics that are discussed in order to explain the relatively low rate of PD observed. The impact of loneliness and social isolation are a major focus for preventive policies based on community resources and early detection of the symptoms of PD.

## Background

Psychological distress (PD) can be defined as a state of poor mental health associated with symptoms from the anxiety-depressive spectrum. Significant levels of psychological distress and other mental health–related problems have been found in college students around the world [1-6], with prevalences often much higher than those found in the general population [1,2,6]. Indeed, autonomy [7,8], logistical independence [4,9], financial difficulties [10] and aspects of academic life such as academic overload, pressure to succeed, competition and uncertainty about the future [1,3,10-12] might act as specific stressful factors in students and then lead to poor mental health. Although PD does not directly reflect a clinical diagnosis [13,14] and does not necessarily require therapeutic or clinical intervention, PD is nevertheless a key subject for primary preventive policies since it could lead to maladaptive behaviours, especially substance use and abuse or misuse of pharmaceutical drugs, and to psychiatric disorders [15-17].

Prevalence rates of PD in students differ considerably from country to country and according to the tools used, ranging from 25.7% in a French sample of 1,723 first-year university students evaluated using the MH-5 scale [18], to 67.4% in an Australian cohort of 6,479 students evaluated using the Kessler-10 screening scale [19] and 83% in an American cohort of 1,773 students [20] evaluated using the Dysphoria Domain of the Trauma Symptom Inventory [21]. In research targeting medical students, Dyrbye et al. [22] found that nearly all (82%) students were experiencing at least one form of distress (such as burnout, depression, stress, mental quality of life (QOL), physical QOL or fatigue), with 58% having at least 3 different forms of distress. In addition, some areas of study could be more at risk than others: Verger et al. asserted that medical studies were a specific risk factor for PD [12].

Besides the expected higher prevalence of PD in women than men [1-3,23], other factors identified as associated with PD in students include psychological factors such as a feeling of lack of mastery [12], recent life events and a psychiatric history [12], plus some situational factors such as being a full-time student, being an undergraduate [23], financial stress [23], academic factors [6] and lack of social support [24-26].

The general applicability of results concerning students’ mental health is limited. Most studies are based on samples that are not representative of the general student population. Most of the samples studied are confined to a single university or a limited number of universities [3-5], to the first or second years of study [3,4], to one academic field - particularly medicine [1,3,4] - or are selected using non-probabilistic methods [1,3,4].

Moreover, most studies have looked into psychological, socioeconomic or sociodemographic factors or life events. Little is known about the impact on student mental heath of more socio-environmental aspects such as academic factors (year of study, area of study) or social participation. To our knowledge, few studies have looked into the impact of academic factors [6] (e.g. year of study, area of study), loneliness [3] or social support, and none has looked into the effects of social participation.

However, a direct link between PD and variables assessing social participation has been shown in the general population. Berry et al. [27] defined as the “big 7” a list of seven factors measuring social participation and social support that reduced the levels of PD in an Australian cohort in New South Wales. The seven factors were: contact with immediate household, extended family, friends, and neighbours, participating in organised community activities, taking an active interest in current affairs, and religious observance. Murray et al. [28], controlling for age, gender and income, found that social participation was also positively associated with positive affects and satisfaction with life, and negatively associated with negative affects. In a Swedish cohort of 51,414 subjects aged 16–84 years, Ahnquist et al. [29] likewise found a negative link between social participation and PD in men (evaluated using GHQ-12 [30]). A link between social participation and psychological distress has also been shown in specific contexts or populations. Indeed, it seems that social participation can act, directly or as a moderator, on the effects of negative life events on mental health in those with a low socioeconomic status and many stressors and in vulnerable people such as disadvantaged ethnic minorities [24-26].

In the context of college, student life considered as a specific period of vulnerability due to the psychological and environmental stressors mentioned earlier, social participation could act as in other vulnerable populations [24-26], working as a protective factor in the prevention of psychological distress and its negative consequences (increased risk of anxiety, depression, substance use and personality disorders, as well as school failure, work difficulties and adverse social consequences later in life [31,32]).

Using a representative sample of French college students, this study aims to investigate the prevalence of and factors associated with PD, and to look more closely at the relative impact of academic and social variables and especially social participation. A focus on these variables should contribute to a better understanding of how PD can develop and provide inspiration for developing health promotion interventions that involve acting on students’ academic and social environment.

## Methods

### Participants

Data were extracted from the 2010 “National Health Barometer”, a large telephone survey of health behaviours in a representative, random sample of the population aged 15–85 years living in France, carried out by the French National Institute of Health Promotion and Health Education (INPES).

Data for the 2010 National Health Barometer were collected between 22 October 2009 and 3 July 2010 by interviewers from a professional survey firm who received specific training for this large nationwide health survey. It was carried out in France using a computer-assisted telephone interview (CATI) system. It allows direct data capture, valuable skip patterns and automatic detection of inconsistencies.

The study protocol included: (a) a formal request to participate, explaining the objectives of the study that was delivered by mail before (or after for subjects with ex-directory numbers whose address was unknown) the first telephone call; and (b) a telephone interview. Unsuccessful calls were repeated 30 and 90 minutes later; up to 40 attempts were made, on different days and at different times. Informed consent was obtained at the start of the telephone interview, in accordance with the guidelines of the French Data Protection Authority (CNIL).

### Sampling

This survey was based on a two-stage random sample of 27,653 French people aged 15 to 85 years speaking French and living in metropolitan France, stratified by region and level of urbanisation. Residents of multi-dwelling buildings, hospitals and institutions were excluded from the target population. This included college dormitories or other common student residential options, but this did not notably affect the general validity of the sample obtained because the vast majority of them could be reached via their parent’s household or on their mobile phone. Private households with landline phones, whether in the telephone directory or not, were included in the sample. People owning only mobile phones were also included (12% of the whole sample, which was the current rate in France in 2009). The first step in sampling was household selection (by phone number), then an eligible subject was selected to answer the questionnaire. This selection was made using the method proposed by Leslie Kish: everybody in the household was first listed, then one of them was selected at random using a CATI computing method [33]. Only one person in each household participated. If a household or respondent refused to participate or could not be contacted, they were not replaced. All collected data were anonymous and self-reported. The mean duration of an interview was about 32 minutes for landline phones and 34 minutes for mobile phones. The overall complete participation and response rate (completed questionnaires only) was 61%. Details of the survey methodology have been published elsewhere [34,35]. This survey was approved by the French Data Protection Authority (CNIL). The final study subsample comprised 5,048 participants aged 18–30 years in whom the analyses were performed, of which 2,860 were working, 666 were unemployed, 576 were in other activities (high school, other non-workers) and 946 were students (college). In the year of the survey, 2.3 million persons were college students in France, so that the sampling rate was 0.04%.

### Instruments

PD was measured using the mental health scale of the SF-36 (MH-5), one of the mental health subscales of the SF-36 [18] scale which has proved useful in screening for psychiatric disorders [36-38]. In this 5-point Likert scale, MH-5 can be used as a categorical instrument with a cut-off point of 56, where lower scores indicate the presence of PD [39].

The main variables of interest that we investigated were:

• Educational variables: type of education, educational level and field of study;

• Opportunity for social contacts, assessed using the 3-point Likert scale (*none, a little, a lot*) and the item “In the last 8 days, did you meet any friends or relatives (including telephone contact)?”;

• Social participation, assessed using the 3-point Likert scale (*none, a little, a lot*) and the item “In the last 8 days, have you had any group or leisure activities such as meetings, religious activities, club activities, going to the theatre, doing sports, going to parties…?”;

• The fact that respondents lived alone in their own home (item: “how many people -including yourself - live in your home?”).

The other variables included as covariables were:

• Sociodemographic variables (age, gender);

• Sexual violence, assessed using two items on a life-time basis (“have you ever been fondled?”; “have you been forced to have unwanted sex?”);

• Nonsexual violence, assessed on the basis of at least one report of theft, threat, a contemptuous look or a slap, that occurred in the last 12 months, collected using multiple-choice questions;

• Life events before age 18, which included serious illness of a parent, death of a parent, violence between parents, parents’ divorce, serious money issues, death of a brother or a sister, collected using multiple-choice questions. In the survey, those events were restricted to the ones that occurred before age 18 because their impact depends very much on the period of life in which they occur [40-42];

• Recent migration concerning people who were born abroad and arrived in France 5 years ago or less.

Sociodemographic data were assessed using the standard sociodemographic questionnaire of the National Health Barometer [43]. A question about regular/occasional active employment was also added for students. It shows that 692 students (73.2%) reported such employment.

### Procedure and data analysis

Data were weighted by the inverse probability of inclusion, i.e. the number of eligible persons divided by the number of telephone lines in the household. They were then adjusted for the target population structure (the 2008 French population structure issued by the National Institute of Statistics and Economic Studies) according to age, gender, region, level of urbanisation and educational level. Calibration weightings were taken from a general regression (GREG), a specific type of calibration adjustment. Analyses were performed using the Stata V10 statistical software package. Firstly, bivariate analyses were made for PD and risk factors, using percentages and Pearson’s chi-squared tests with Rao-Scott second order correction (F statistic). The numbers shown were unweighted, but the percentages were weighted.

Then logistic regressions for the variables of interest (academic and social) that were associated with PD at the 0.05 level in bivariate analyses were computed, adjusted for sociodemographics and life events.

## Results

In the whole sample of participants aged 18–30 years, the PD rate was 13.8% in students. It was 12.6% in working people and 23.4% in unemployed people.

Table 1 shows the bivariate analyses (F statistic and P from the design-based Pearson’s chi-squared test) for social and educational factors, life events and sociodemographic data and levels of PD in students, together with adjusted regression analyses (odd ratios and 95% CI).

**Table 1:** Bivariate analyses and multivariate analyses of life events and sociodemographic, educational and social factors in students

			**Bivariate analyses**		**Logistic regression**	
	**N**	**MH5 < 56 (%)**	**F**	**P**	**OR**	**95% CI**
	**946**	**13.8**				
**Sociodemographics**						
*Gender*			24.077	<0.001		
Male	427	7.2			1	REF
Female	519	19.5			2.6***	1.6-4.2
*Age*			1.668	0.172		
18-19	248	10.0			1	REF
20-21	330	14.4			1.4	0.8-2.6
22-23	206	14.2			1.8	1.0-3.5
>23	162	19.1			1.5	0.8-3.1
*Work*			3.428	0.033		
Regular employment	201	15.6			1	REF
Occasional employment	491	10.8			0.7	0.4-1.2
No employment	251	18.5			1.0	0.6-1.8
*Income received*			12.201	<0.001		
Comfortable	227	8.5			1	REF
I’m ok	433	10.0			0.9	0.5-1.6
I can make it with difficulties	196	21.7			1.5	0.8-2.8
Difficult/Debts	89	33.4			2.4*	1.2-4.9
**Life events**						
*Life events before 18*			11.749	<0.001		
None	523	10.2			1	REF
At least one	414	19.0			1.4	0.9-2.1
*Nonsexual assault in the last 12 months*			17.989	<0.001		
No	600	9.8			1	REF
Yes	346	21.0			2.8***	1.9-4.3
*Lifetime sexual assault*			3.755	0.053		
No	877	13.2			1	REF
Yes	69	22.7			1.2	0.6-2.3
*Recent migration*			1.046	0.307		
No	882	13.5			1	REF
Yes	64	19.0			0.9	0.4-2.1
**Educational variables**						
*Type of education*			2.502	0.043		
Preparatory class	47	17.8			1.6	0.6-3.9
Vocational training	228	12.6			0.8	0.5-1.5
University	425	15.4			1	REF
Engineering or commercial school	121	4.8			0.4	0.2-1.0
Other	122	17.6			1.1	0.5-2.0
*Field of study*			2.357	0.022		
Sciences	227	10.3			1	REF
Humanities	173	15.0			0.9	0.4-1.7
Law	63	9.1			0.3*	0.1-1.0
Economics	218	14.3			0.9	0.5-1.8
Sports science	22	9.3			0.7	0.1-3.8
Medicine	143	15.5			0.7	0.3-1.5
Arts	44	32.3			2.2	0.9-5.3
Others	41	6.5			0.8	0.2-3.0
*Educational level*			0.778	0.539		
1 year	294	10.7				
2 years	244	14.9				
3 years	168	15.6				
4 years	118	14.8				
5 years +	122	16.4				
**Social variables**						
*Lives alone*			4.187	0.041		
No	666	12.8			1	REF
Yes	280	18.6			0.9	0.6-1.5
*Contact with friends and family*			1.266	0.282		
None	128	18.7				
A little	357	12.4				
A lot	461	13.5				
*Social activities*			15.099	<0.001		
None	112	28.1			4.1***	2.2-7.5
A little	386	17.4			2.3**	1.4-3.6
A lot	448	7.5			1	REF

*p < .05, **p < .01, ***p < .001.

### Sociodemographic factors

High variations in the frequency of PD were found in our sample between male (7.2%) and female (19.5%) participants (OR = 2.6, 95% CI = 1.6 to 4.2; p < .001).

Age did not appear to be significantly associated with frequency of PD, and employment status did not remain significant when adjusted for covariates. Low income was associated with a higher level of PD: 33.4% of students who reported financial difficulties or debts were classified as having PD vs. 8.5% of students who said they were “comfortable” (OR = 2.4, 95% CI = 1.2 to 4.9; p < .05).

### Life events

Nonsexual assault in the last 12 months was highly associated with PD (21.0% vs. 9.8%; OR = 2.8, 95% CI = 1.9 to 4.3; p < .001), while sexual violence, recent migration and presence of an adverse life event before the age of 18 were unassociated with PD after adjustment.

### Educational and social factors

Educational level did not appear to be significantly associated with the frequency of PD. The type of education was significantly correlated with PD in the bivariate analysis but did not remain significant when adjusted for covariables. Field of study was significantly associated with the frequency of PD in the bivariate analysis and remained significant after adjustment. Studying law was associated with a lower level of PD: 9.1% of those who said they were studying law were classified as having PD vs. 13.8% in the whole student population (OR = 0.3, 95% CI = 0.1 to 1.0; p < .05).

Living alone was not related to PD after adjustment. High social participation, i.e. reporting a high level of participation in social and/or community activities, was associated with a lower level of PD (7.5% vs. 17.4% in students who said they participated *a little*, OR = 2.5, 95% CI = 1.4 to 3.6; p < .01, and 28.1% in participants who said they did not participate, OR = 4.1, 95% CI = 2.2 to 7.5; p < .001). Contact with friends or family in the last week was not associated with PD.

## Discussion

The results of this study revealed that, within a representative sample of French students, the prevalence of PD is not as high as expected (13.8%) when assessed with the MH-5 scale. Moreover, we did not find a higher prevalence of PD in young or first-year students. This contrasts with several studies which suggested that students were a vulnerable population with high rates of PD (e.g. [22]), specifically in first-year students [12]. French students are a specific population in terms of socioeconomic characteristics. Public academic studies cost about € 500 a year, so that a high proportion of young adults of the same age can benefit from university curricula without taking out loans, as can be observed in many other national contexts. Indeed, the low proportion of students reporting debts or being in financial difficulty (the latter being significantly more at risk of psychological distress) could partly help reduce the overall prevalence of psychological distress found in our sample of French students.

While we found that, as per the literature, violence in the last year was highly related to psychological distress, lifetime sexual assault was not significantly associated with our dependent variable, although a clear trend was identifiable in the bivariate analyses (22.7% of participants who had been a victim of sexual violence in life reported PD, compared with 13.5% of those who had not been a victim of such assaults; F = 3.7; p = .05); this link became non-significant when controlled for other variables. Co-variation with other independent variables (gender and social isolation) might partially explain this finding. The limited number of variables used to define the process by which lifetime sexual violence affects the expression of distress in students does not allow us to draw any firm conclusions from this study.

Although expected, a link between contacts with friends and family and psychological distress was not observed. Several interpretations might explain this lack of connection. On the one hand we can assume that the measurement was not sufficiently specific (friends and family were combined, as were telephone contacts and physical ones). On the other hand, whereas the quantity of contacts was assessed, no account was taken of their quality. Finally, the geographical coverage of universities in France may be sufficiently dense for students to be able to live closer to their family and social network of friends. However, the limits of our measurement of *contacts with friends and family* mentioned above do not allow us to draw any firm conclusions from this result.

The higher prevalence notably found by Verger et al. [12] in a French student sample by comparison with our study could be explained mainly by a difference in the assessment of psychological distress and to a lesser extent by a difference in sample structure. Verger et al. used the first version of the MH-5 (cut point at 52 with a 6-point scale) whereas we used, as in the 2010 National Health Barometer secondary analyses, a second version of the MH-5 with a cut point at 56 (and a 5-point scale) [38]. This does not allow us to make any comparison between these two studies. Moreover, the sample used by Verger et al. consisted exclusively of first-year students from only public universities, excluding about 30% of young adults studying in private schools. However, conditions of education, social and professional opportunities are more uncertain in French public universities than in engineering or business schools, where more than 8 out of 10 students find a job within a year after completing their studies. On the basis of our results which show a very low proportion of psychological distress in engineering or business schools, the inclusion of these students in our survey might also have helped to reduce the overall prevalence of psychological distress in our sample.

Consistently with previous research (e.g. [12,23,44]), we identified that, after controlling for all variables, gender (OR in female students = 2.6), financial stress (OR = 2.4) and nonsexual violence in the past year (OR = 2.8) were associated with PD. The effect of medicine as field of study, which was identified by Verger et al. as a risk factor for PD [12], was not detected in our study, whereas participants studying law were less at risk of PD (OR = 0.3). This could be at least partly due to the parents’ socioeconomic status which is higher in French law students by comparison with other fields of study. Finally, as expected we found that low participation in social activities (the item was: “In the last week, have you had any group or leisure activities such as meetings, religious activities, club activities, going to the theatre, doing sports, going to parties…?”) was associated with a higher level of PD, starting with students who said they did not participate in any group or leisure activities (OR = 4.1) to students who said they participated *a little* (OR = 2.3), compared with those who said they participated *a lot*.

Participation in social activities is thus the biggest predictor of PD that we observed in our study. This finding adds to the results previously published on the general population [29] or on disadvantaged ethnic minorities [24-26] the fact that students are also and even particularly likely to benefit from a programme using social participation as a lever of prevention and health promotion. This offers opportunities for developing actions aimed at promoting group or leisure activities as a general preventive instrument in student populations.

Recently, a number of local initiatives have been set up in France to address social isolation in students. Social meetings initiatives, world cafés aimed at providing meetings with mental health practitioners and hence at reducing the reluctance to ask for counselling or help, local training for community leaders in visiting isolated students or door-to-door visiting by psychology students have been initiated (see http://www.fage.org, http://apsytude.e-monsite.com), but have not yet been assessed.

## Conclusions

To conclude, our results suggest that social participation should be considered a key factor in further research into the development of preventive interventions for students. Work on social participation seems to be promising when targeted at students who have to spend a lot of time in common and collective spaces (campus, classrooms, halls, cafeterias) that are particularly convenient for the development of participative projects and actions. Combating loneliness and promoting collective initiatives may result in improved mental health within student communities. Students’ social environment (i.e. campus with multiple facilities) should make possible local initiatives that promote social participation.

These local initiatives should pay special attention to women and to students with financial difficulties, both of whom have higher rates of PD. It has been shown that financial stress in students often comes with poorer housing conditions and social isolation. Working on this specific subgroup together with social workers would allow poorer students and need-based scholarship holders to be targeted for specific housing and socially-based preventive services aimed at preventing the mental health consequences of poor living conditions, together with services aiming at promoting better living conditions. As an example, social groceries have been appearing recently in France (see http://www.gaelis.fr) to support students on low income by offering low-cost essential foods and developing social events for every student living on the campus.

As regards strengths and limitations, the main strength of our findings is that they are based on a random sample that is representative of the general French student population, including all years of study and a large variety of academic fields. Several limitations however deserve attention in the interpretation of the findings. First, the response rate obtained in the survey was 61%, which is satisfactory for such health surveys in France, but lower than the rates obtained in other epidemiological mental health surveys such as the NCS-R [45] or the NESARC [46]. Moreover, focusing on current college students, we can underline the fact that PD is a risk factor for dropping out of college [47,48] which may skew our estimates. The limited number of variables used in this study demand the inclusion, in future surveys of the student population, of more control variables (such as depression) and the expansion and refinement of variables assessing social support and social participation.

## Competing interests

The authors declare that they have no conflicting interests.

## Authors’ contributions

Devised and designed the experiments: TS EDR LV FS FB. Performed the experiments: TS EDR RG JBR SL FB. Analysed the data: TS EDR LV RG JBR SL FS VK FB. Contributed reagents/materials/analysis tools: TS EDR LV RG JBR SL FS VK FB. Wrote the paper: TS EDR RG FB. All authors read and approved the final manuscript.

## Pre-publication history

The pre-publication history for this paper can be accessed here:

http://www.biomedcentral.com/1471-2458/14/256/prepub
